# Synergistic effect of corn straw and mulberry leaves on *in vitro* fermentation, growth performance and antioxidant capacity of sheep

**DOI:** 10.5713/ab.24.0763

**Published:** 2025-02-27

**Authors:** Qirui Hou, Wenfeng Zhang, Shuli Wang, Wenyu Hou, Weiguo Zhao, Hui Xie, Peng Zhao, Yongen Zhang

**Affiliations:** 1 Jiangsu Key Laboratory of Sericultural Biology and Biotechnology, School of Biotechnology, Jiangsu University of Science and Technology, Zhenjiang, China; 2 Key Lab of Agricultural Outlook, Ministry of Agriculture and Rural Affairs of the People’s Republic of China, Beijing, China; 3 School of Biological Engineering, Xinxiang University, Xinxiang, China; 4 Henan Yunfei Science and Technology Develop Co., Ltd., Zhengzhou, China; 5 Henan Sericulture Research Institute, Zhengzhou, China; 6 Agricultureal Informantion Institure of Chinese Academy of Agricultural Sciences, Beijing, China

**Keywords:** Antioxidant, Fermentation, Growth Performance, Meat Quality, Synergistic Effect

## Abstract

**Objective:**

This study aimed to investigate the synergistic effects of corn straw and mulberry leaves on the growth performance and antioxidant capacity of sheep.

**Methods:**

Corn straw and mulberry leaves were mixed in six different ratios as culture substrates: 100:0 (T0), 80:20 (T20), 60:40 (T40), 40: 60 (T60), 20:80 (T80) and 0:100 (T100). Rumen fluid from small-tailed Han sheep was collected, and artificial saliva was prepared. Gas production (GP) parameters, volatile fatty acids (VFAs), ammonia nitrogen (NH_3_-N), microbial crude protein (MCP) and additional indicators of *in vitro* rumen fluid fermentations were measured over 72 h. Combinations with a positive multiple-factors associative effect index were chosen for a 65 d *in vivo* feeding experiment, during which the growth performance, carcass traits, meat quality, and liver antioxidant function were evaluated.

**Results:**

Mulberry leaves exhibited a positive correlation with *in vitro* theoretical maximum GP, cumulative GP, MCP, and organic matter digestibility. At 72 h, total VFA and acetic acid were significantly higher in the T60, T80, and T100 groups (p<0.05). Additionally, at 6 h, 12 h, and 72 h, the NH3-N of the T60, T80, and T100 groups were significantly higher (p<0.05). The T20, T60, and T80 combinations were selected for sheep feeding, and T60 got the highest increase in average daily feed intake (p<0.05). Moreover, the thickness of the rib and back muscle were notably increased in the T60 and T80 groups (p<0.05). Water-holding capacity of the *longissimus dorsi* muscle was higher and drip loss was lower in the combination-fed groups, particularlly in the T20 and T60 groups (p<0.05). Blood urea nitrogen levels decreased with increasing mulberry leaf content (p<0.05). Furthermore, the T60 and T80 groups demonstrated higher α,α-diphenyl-β-picrylhydrazyl and 2,2′-Azino-bis(3-ethylbenzothiazoline-6-sulfonic acid) free radical scavenging ability (p<0.05). Catalase and glutathione peroxidase activities in the sheep liver were also notably enhanced (p<0.05).

**Conclusion:**

The combination of corn straw and mulberry leaves had a positive synergistic effect on improving feed intake, muscle thickness, muscle water retention, and the liver antioxidant capacity in sheep. The ratios of 40:60 corn straw to mulberry leaves showed the most beneficial effects. This combination provides a practical method for utilizing agricultural waste and optimizing sheep diets.

## INTRODUCTION

There are abundant crop straw resources in developed planting areas. However, the low crude protein (CP) content and digestibility of crop straw can impede livestock digestion and nutrient absorption. Corn straw is a common coarse fodder in ruminant diets, but it has a low CP content and suboptimal utilization rates [1]. Therefore, enhancing the utilization efficiency of roughage has emerged as a pivotal focus within the domain of feed research.

Mulberry (*Morus alba* L.) is a perennial deciduous woody plant that is widely distributed between 10 degrees south latitude and 50 degrees north latitude. Mulberry leaves serve as the primary food sourcefor silkworms. When the silk industry declined, a large number of mulberry leaves were abandoned and underutilized. Fortunately, mulberry leaves are rich in nutrients such as protein and amino acids, as well as antioxidant compounds such as flavonoids and polysaccharides, which have high feeding value and were included in the “Feed Raw Materials Catalogue” by the Ministry of Agriculture of the People’s Republic of China in 2012 [2]. Further research has revealed that mulberry leaves contain alkaloids, sterols and other beneficial components that can help lower blood sugar levels and reduce blood lipids [3]. Unfortunately, these components may not be conducive to animal fattening, thereby limiting their use in animal feed. Studies have demonstrated that microbial fermentation can effectively reduce the levels of anti-nutritional factors in mulberry leaves while enhancing CP content [4]. Therefore, mulberry leaves hold great potential for used in ruminant feed.

It is imperative to investigate the nutritional value of feed and the synergistic effects between different feeds [5]. When a specific combination of feeds leads to enhanced feed utilization or intake indicates a positive synergistic effect. For instance, mixing corn straw with amaranth in silage has been demonstrated to improve fermentation quality, aerobic stability, and rumen degradation [6]. Moreover, combining corn and wheat straw has been shown to enhance the nutritional value of paper mulberry silage by reducing mycotoxin production, pH levels, and ammonia nitrogen (NH_3_-N) content while increasing lactic acid production [7]. Research has been conducted on the fermentation of rice straw and mulberry leaves [8], as well as on the fermentation of corn straw and paper mulberry. However, there is currently a lack of studies examing the fermentation effects of corn straw and mulberry leaves.

This study investigates the synergistic effects of varying proportions of corn straw and mulberry leaves during *in vitro* fermentation. Subsequently, the optimal ratio was determined for feeding trial to evaluate the impact of this synergistic combination on the growth performance and antioxidant capacity of sheep. The aim is to identify a rational utilization of agricultural waste and reduce feeding costs.

## MATERIALS AND METHODS

### Animal ethics

The animal handling protocols, experimental designs, and methodologies were approved by the Animal Care and Use Committee of Jiangs University of Science and Technology, Zhenjiang, Jiangsu province, China (ethical approval number: GQ20231117).

### Rumen fluid collection and artificial saliva preparation

Four healthy male small-tailed Han sheep, each weighing 16.7±0.6 kg and equipped with permanent rumen fistulas, were chosen as donors of rumen fluid. The sheep were fed oat grass supplemented with a corn-to-soybean mixture (6:4 ratio) twice daily (at 8:00 am and 6:00 pm) for two weeks. Animals had free access to water. Rumen liquid was collected into sterile homemade vacuum negative pressure devices immediately before the morning feeding. The liquid was promptly filtered and transferred into a preheated flask placed in a water bath maintained at 39°C and filled with CO_2_.

The artificial saliva used for this experiment: 8.75 g of NaHCO_3_, 1.00 g of NH_4_HCO_3_, 1.43 g of Na_2_HPO_4_, 1.55 g of KH_2_PO_4_, 0.15 g of MgSO_4_·7H_2_O, 0.52 g of Na_2_S, 0.015 g of MnCl_2_·4H_2_O, 0.002 g of CoCl_2_·6H_2_O, 0.012 g of FeCl_3_·6H_2_O, 0.017 g of CaCl_2_·2H_2_O and 1.25 mg of resazurin in 1 L of distilled water, resulting in a pH of 6.8.

### *In vitro* gas production and batch culture

The corn straw used in this experiment was provided by Zhenjiang Zhongrun Feed Co., LTD, while the mulberry leaves were provided by the Sericulture Institute of Chinese Academy of Agricultural Sciences. All samples were dried at 65°C and subsequently passed through a 40-mesh sieve. The corn straw and mulberry leaves were mixed in varying proportions to form six treatment groups: 100% corn straw (T0 group), 80% corn straw with 20% mulberry leaves (T20 group), 60% corn straw with 40% mulberry leaves (T40 group), 40% corn straw with 60% mulberry leaves (T60 group), 20% corn straw with 80% mulberry leaves (T80 group), and 100% mulberry leaves (T100 group). The nutritional components were determined according to AOAC guidelines [9]. The nutrient levels of the substrates in each group are presented in Table 1. Three replicates were measured and expressed as average values.

Each of the six substrates (T0, T20, T40, T60, T80, and T100) was accurately weighed to 0.5 g and transferred into a preheated 100 mL fermentation flask maintained at 39°C. Each group included 7 replicates. 50 mL of preheated artificial saliva and 25 mL of rumen fluid were rapidly added to the fermentation flask, followed by the injection of CO_2_ for approximately 5 s before sealing the flask with a rubber stopper. The fermentation flask was then connected to the gas pathway of a 64-channel AGRS-III type *in vitro* fermentation devices for automatic gas production (GP) recording. The mixture was allowed to ferment continuously for 72 h at 39°C. GP parameters, including the theoretical maximum GP at 72 h, potential GP, and the constant of GP rate, were detected. Cumulative GP was calculated using the Ørskov and McDonald model [10].


$$GP_{t}=a+b\times (1-e^{-ct})$$

where, *GP_t_* represents the cumulative GP at t time point (mL/g dry matter [DM]); *a* represents the rapid GP component, that is, the GP at the initial fermentation time point (mL/g DM); *b* represents the slow GP component, that is, the theoretical maximum GP (mL/g DM); *c* is the *in vitro* fermentation gas production rate constant (mL/h), and *a*+*b* is the potential GP (mL/g DM).

In the *in vitro* batch culture experiment, artificial saliva, and the weighed substrate were prepared as described earlier. Each group consisted of 18 replicates and was cultured at 39°C for 72 h in a thermostatic oscillating water bath (SHA-A; Changzhou Jiebosen Instrument Co., LTD, Changzhou, China). Samples were collected at 3, 6, 12, 24, 48, and 72 h post-culture, with three fermentation bottles sampled from each group. The fermentation products were filtered through 4 layers of gauze. The filtrate was divided into two 10 mL centrifuge tubes and three 2 mL centrifuge tubes, then stored at −80°C for subsequent determination of volatile fatty acids (VFAs), NH_3_-N, and microbial crude protein (MCP). The residue was washed and dried to determine *in vitro* organic matter digestibility (IVOMD). VFAs levels were measured using gas chromatography (Agilent 7890A; Agilent Technologies, Santa Clara, CA, USA) following the method described by Khorasani et al [11]. The pH was measured using an electrode pH meter (BPH-7200; Beijing Zhongheng Rixin Technology Co., Ltd., Beijing, China). NH_3_-N concentration was determined using a spectrophotometer (Agilent Cary 7000; Agilent Technologies) according to the method outlined by Broderick and Kang [12]. MCP concentration was measured using the Kjeldahl nitrogen determination method (FOSS Kjeltec 8400; FOSS, Mulgrave, Australia). IVOMD was estimated using the combustion method according to the following formula:


$$\frac{\text{Substrate organic content}-(\text{Residual organic content}-\text{Blank organic content})}{\text{Substrate organic content}}\times 100\%$$

The formulas for evaluating the combined effect were as follows:

Weighted value (WV) = Value of T0 group×Corn straw proportion+Value of T100 group×Mulberry leaves proportion


$$\text{Single-factor associative effect index }(\text{SFAEI})=\frac{\text{Measured value}-\text{WV}}{\text{WV}}\times 100\%$$

Multiple-factors associative effect index (MFAEI, %) = ∑SFAEI = SFAEI _Cumulative GP_+SFAEI _IVOMD_+SFAEI _MCP_+SFAEI _NH3-N_+SFAEI _TVFA_

### Animals and diets

Thirty-two healthy male small-tailed Han sheep, approximately four months old and weighing 24.14±0.78 kg, were utilized in the feeding experiment. The sheep were randomly assigned to four groups, with eight sheep in each group. The experimental diets included three combinations of corn straw and mulberry leaves: 80% corn straw and 20% mulberry leaves (T20 group), 40% corn straw and 60% mulberry leaves (T60 group), and 20% corn straw and 80% mulberry leaves (T80 group), with solo corn straw as the control. Each combination constituted 40% of the total diet. The chemical composition and nutritional content of the feed are presented in Table 2. The compositions were analyzed following AOAC methods [9], ensuring that the nutrient content aligns with the requirements of NRC [13]. The experiment lasted for 65 d, with a 10 d adaptation period before the start of the experiment. The sheep were housed in individual pens and fed twice daily at 07:30 am and 05:00 pm. Water was provided *ad libitum*, and the animals were exposed to standard lighting conditions.

### Growth performance, serum metabolites and carcass traits

During the formal trial period from 11 to 65 d, both the feed offered and refused were weighed daily to calculate the average daily feed intake (ADFI). The sheep were weighed at the beginning and end of the experiment to determine the initial live weight (LW), final LW, and average daily gain (ADG). The feed-to-gain ratio (F/G) was calculated as the ratio of ADFI to ADG.

After the feeding experiment, four sheep from each group were randomly selected for an overnight fast. The following morning, whole blood samples were collected prior to slaughter. The blood samples were allowed to clot at room temperature for 6 h, after which they were centrifuged at 3,000×g for 15 min to obtain serum. Serum metabolites, including aspartate aminotransferase (AST), alanine aminotransferase (ALT), total protein, albumin, globulin, creatinine, blood urea nitrogen (BUN), uric acid, glucose, total cholesterol, triglyceride, high-density lipoprotein cholesterol and low-density lipoprotein cholesterol were analyzed using automatic biochemical analyzer (HITACHI 7080; Hitachi, Tokyo, Japan) according to the methods described by Hsu et al [14].

Carcass weight was measured after removing non-carcass components on the day of slaughter. The carcasses were then stored at 4°C for 48 h. During the slaughtering process, the carcasses were cut between the 12th and 13th ribs to assess subcutaneous fat thickness [15], rib meat thickness, and back meat thickness [16]. The remaining carcasses were used to measure the eye muscle area, abdominal fat, pericardial fat, perirenal fat, and tail fat [17]. Liver tissues were collected, divided into centrifuge tubes, quickly frozen in liquid nitrogen, and transferred to a −80°C freezer for storage.

### Meat quality

The left side muscles of the *longissimus dorsi* (LD), anterior leg (AL), and hind leg (HL) were sampled for the evaluation of meat quality. Prior to measurement, the samples were allowed to equilibrate at room temperature for 30 min to facilitate the formation of oxymyoglobin. The Minolta CR-400 colorimeter (Minolta, Osaka, Tokyo) was used to measure meat color at three points on the carcass using the CIE (L*, a*, and b*) system, and the average readings were recorded. Water-holding capacity (WHC) was determined according to the protocol outlined by Farouk and Wieliczko [18]. Drip loss (DL) was measured following the procedure described by Wang et al [19]. Cooking loss was assessed by submerging the samples in a water bath maintained at 75°C for 90 min, followed by cooling to room temperature and dring with paper towels. Warner-Bratzler shear force (WBSF) was evaluated using a Lloyd TA1 texture analyzer, following the method described by Hopkins et al [20].

### Analysis of liver antioxidant function

A 0.05 g liver sample was homogenized using a FSH-2 homogenizer (Ronghua Instrument Manufacture Co., Ltd., Changzhou, China) in a test tube with 2 ml of ice-cold 120 μM α,α-diphenyl-β-picrylhydrazyl (DPPH) in methanol. The homogenate was then incubated at room temperature for 30 min in the dark and subsequently centrifuged at 1,400×g for 10 min using a Sigma 3K15 centrifuge. The absorbance of the supernatant was measured at 517 nm using a spectrophotometer (model 752; Shanghai Spectral Instrument Co., Ltd., Shanghai, China). Finally, the activity was calculated using the following equation:


$$\text{DPPH free radical scavenging activity}(\%)=({\text{Abs}}_{\text{control}}-{\text{Abs}}_{\text{sample}})/{\text{Abs}}_{\text{control}}\times 100$$

A solution was prepared by mixing 7 mM 2,2′-azinobis(3-ethylbenzthiazoline-6-sulphonate) (ABTS) with 2.45 mM K_2_S_2_O_8_ and incubating in the dark at room temperature for 16 h. Then the solution was diluted with 80% ethanol until an absorbance of 0.700±0.005 at 734 nm was achieved. A 0.05 g liver sample was homogenized with 4 mL of the diluted ice-cold ABTS^+^ solution. The mixture was incubated at room temperature in the dark for 30 min and subsequently centrifuged at 1,400×g for 10 min. The absorbance of the supernatant at 734 nm was immediately measured using a spectrophotometer. The ABTS free radical scavenging activity was then calculated using the following equation:


$$\text{ABTS free radical scavenging activity}(\%)=({\text{Abs}}_{\text{control}}-{\text{Abs}}_{\text{sample}})/{\text{Abs}}_{\text{control}}\times 100$$

The levels of reactive oxygen species (ROS) were measured using a commercially available kit (Cat NO. E004-1-1; Nanjing Jiancheng Bioengineering Research Institute Co., Ltd., Nanjing, China). A 0.5 g sample was homogenized with 2.5 mL of ice-cold 0.2 M phosphate buffer (pH 7.4). The homogenate was then centrifuged at 1,200×g for 10 min. The resulting supernatant was collected, and 190 μL was added to a 96-well plate. Subsequently, 10 μL of 1 mM dichlorofluorescein diacetate was added, and the mixture was incubated at 37°C for 30 min. The fluorescence intensity was measured using a microplate reader (BioTek Instruments Inc., Winooski, VT, USA) with excitation and emission wavelengths of 485 nm and 525 nm, respectively.

The activities of antioxidant enzymes, including glutathione peroxidase (GPX; Cat NO. JEB-15495), superoxide dismutase (SOD; Cat NO. JEB-15498), catalase (CAT; Cat NO. JEB-15496), and the levels of malondialdehyde (MDA; Cat NO. JEB-15497), were assessed using commercial kits (Jin Yibai Biological Technology Co., Ltd., Nanjing, China) according to the manufacturer’s instructions. To begin, liver tissue was homogenized in phosphate buffer saline (pH = 7.4) and then centrifuged at 1,000×g for 20 min. The resulting supernatants were collected and used as samples. Next, 50 μL of either the standard or sample was added to each well of a 96-well plate and incubated for 30 min at 37°C. The liquid was then removed, and each well was washed five times with 200 μL washing buffer. Subsequently, 50 μL of horseradish peroxidase-conjugated reagents were added to each well and incubated for an additional 30 min at 37°C. Following this, the plates were rewashed five times. Then, 50 μL of chromogen solution A and 50 μL of chromogen solution B were added to each well and incubated for 15 min at 37°C. Finally, 50 μL of stop solution was added to each well, and the absorbance at 450 nm was measured using a microplate reader.

### Data processing and analysis

The test data were compiled using Excel 2021, and figures were generated using GraphPad Prism software (GraphPad Software, San Diego, CA, USA). Data are presented as the mean values±standard errors of the mean and were analyzed using one-way analysis of variance with a Turkey post hoc test in SPSS version 22.0 (IBM Corp., Armonk, NY, USA). All data were checked for normality. For parameters that did not follow a normal distribution, differences between groups were analyzed using the Mann-Whitney U test. When significant differences were detected, multiple comparisons were conducted using Duncan’s test. In this study, the diet was treated as the main effect, and the individual sheep served as the experimental units. A significance level of p<0.05 was considered statistically significant.

## RESULTS

### Synergistic effects of corn straw and mulberry leaves on the gas production parameters of *in vitro* fermentation

As the proportion of mulberry leaves increased, the theoretical maximum GP, potential GP, and cumulative GP at 72 h, all demonstrated a steady increase. The T60, T80 and T100 groups showed significantly higher values compared to the Control (p<0.01), with T100 achieving the highest levels. Additionally, the T60 group demonstrated the highest constant rate of GP (p<0.01). Further details are provided in Table 3.

### Synergistic effects of corn straw and mulberry leaves on the volatile fatty acids concentration of *in vitro* fermentation

As shown in Table 4, with the extension of incubation, the concentrations of acetic acid, propionic acid, and butyrate acid in the culture medium of each group generally exhibited an increasing trend. The concentrations of acetate and total volatile fatty acid (TVFA) in the T80 group were significantly higher than those in the other groups at 12 h and 48 h (p<0.05). At 72 h, there was no significant difference in acetate and TVFA concentrations among the T60, T80 and T100 groups, but these concentrations were significantly higher than those in the other groups (p<0.05). At 12 h, 24 h, and 48 h, the concentration of propionate in the T80 group was significantly higher than that in other groups (p<0.05). At 12 and 72 h, the acetate/propionate ratio in the T60 and T80 groups was significantly higher than that in other groups (p<0.05). Butyric acid was detected in all groups but showed no significant correlation with the content of corn straw and mulberry leaves in the substrate.

### Synergistic effects of corn straw and mulberry leaves on the pH, NH_3_-N, microbial crude protein and IVOMD concentration of 72 h *in vitro* fermentation

As can be seen from Table 5, the pH of the culture solution exhibited a decreasing trend over time. There was no statistically significant differences among the groups, with all pH values falling within the range of 6.5 to 7.0 after 24 h of fermentation. At 3 h, the NH_3_-N concentration in the T40, T60, T80, and T100 groups was significantly higher than that in the T0 group (p<0.05). Subsequently, at 6, 12, and 72 h, the NH_3_-N concentration in the culture medium of the T60, T80, and T100 groups were significantly greater than that of the T0, T20, and T40 groups (p<0.05). The MCP concentration in the culture medium of the T60, T80, and T100 groups was significantly higher than that in the other groups at 12 h and thereafter (p<0.05). In addition, IVOMD showd a growth trend as time progressed,and the proportion of mulberry leaves increased. Specifically, at 6, 24, 48, and 72 h, the IVOMD in the T100 group was notably higher than the others (p<0.05).

As shown in Table 6, at 72 h of fermentation, the cumulative GP, TVFA, MCP, and NH_3_-N in T40 group exhibited adverse combined effects, with all combinations showing a negative SFAEI in cumulative GP. The SFAEI of MCP, TVFA, and NH_3_-N in the T60 group was higher compared to the other groups, whereas the SFAEI of IVDOM decreased as the proportion of mulberry leaves increased. Additionally, the MFAEI in the T60 group was higher than the other groups, followed by T20 and T80.

### Synergistic effects of corn straw and mulberry leaves on animal growth performance and carcass traits

There was no significant impact on the growth rate of sheep (Table 7); however, the ADFI increased after incorporating a combination of corn straw and mulberry leaves into the diet, with the T60 group exhibiting a significantly higher ADFI compared to the Control group (p<0.05). Most carcass traits remained unaffected by the inclusion of mulberry leaves in the diet. Notably, the thicknesses of the rib and back were significantly greater in the T60 and T80 groups compared to the Control group (p<0.05). There were no significant differences in subcutaneous fat, abdominal fat, pericardial fat, perirenal fat, tail fat, and pot fat among the different groups.

### Synergistic effects of corn straw and mulberry leaves on meat quality

Results in Table 8 suggested that the combination of corn straw and mulberry leaves had no significant effect on the meat color (L*, a*, and b*) or WBSF of the LD, AL, and HL muscles when compared to the Control group. The WHC of these muscles were lower in the Control group than in the T20 and T60 groups. The DL of the LD muscle in the Control group was notably higher than in the other groups (p<0.05). Additionally, the CL of the LD muscle in the T20 group was significantly lower than in the other groups, whereas the CL of the HL muscle in the Control group was notably higher than in the T20 and T80 groups (p<0.05).

### Effects of varying proportions of corn straw and mulberry leaves on hematology analysis

As shown in Table 9, it was observed that as the proportion of mulberry leaf in the diet increased, the serum ALT and AST activities in the sheep decreased. Specifically, the ALT and AST activities in the T60 and T80 groups were significantly lower than those in the Control and T20 groups (p<0.05). However, there were no significant differences observed in AST/ALT ratios. Additionally, the serum BUN content in the sheep decreased with the increase in mulberry leaf content. The serum BUN content in the T20, T60, and T80 groups was significantly lower than that in the Control group (p<0.05). Notably, mulberry leaves did not have any significant effects on other indicators of liver function, renal function, or lipid levels in the serum.

### Liver antioxidant function

**Figure 1:**
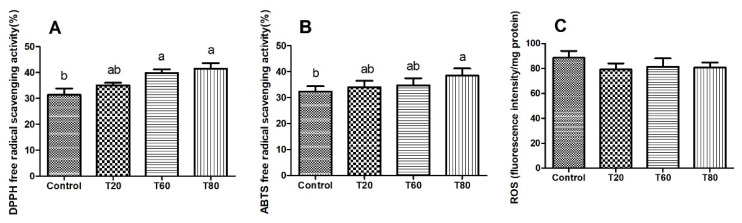
Effect of varying proportions of corn straw and mulberry leaves on DPPH (A) and ABTS (B) free radical scavenging abilities and sheep liver ROS levels (C). Control, 100% corn straw; T20, 80% corn straw with 20% mulberry leaves; T60, 40% corn straw with 60% mulberry leaves; T80, 20% corn straw with 80% mulberry leaves. Values are means and standard deviations represented by vertical bars (n = 4). Bars with unlike letters indicate a significant difference (p<0.05). DPPH, α,α-diphenyl-β-picrylhydrazyl; ABTS, 2,2′-Azinobis-3-ethylbenzthiazoline-6-sulphonate; ROS, reactive oxygen species.

As the proportion of mulberry leaves in the diet increased, the liver of sheep exhibited enhanced DPPH and ABTS free radical scavenging abilities. Specifically, the T60 and T80 groups demonstrated significantly higher DPPH free radical scavenging ability compared to the Control group (Figure 1A), while the T80 group showed significantly greater ABTS free radical scavenging ability (Figure 1B, p<0.05). However, fluorescence probe detection indicated no significant differences in ROS levels among the groups (Figure 1C).

**Figure 2:**
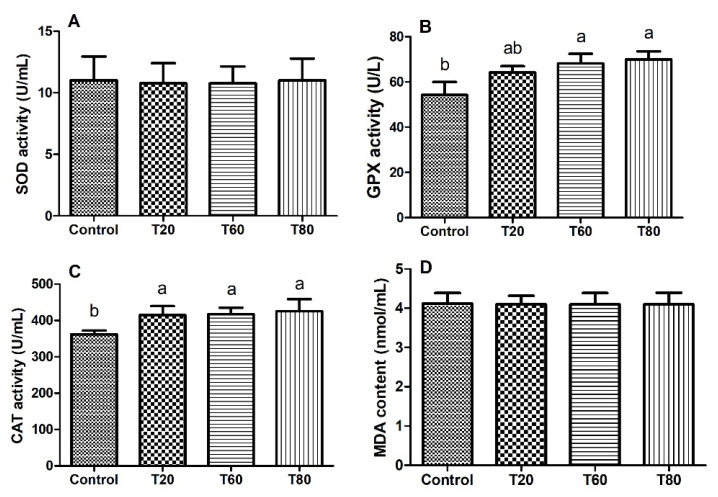
Effect of varying proportions of corn straw and mulberry leaves on SOD (A), GPX (B) and CAT (C) activies, and MDA content (D) of sheep liver. Control, 100% corn straw; T20, 80% corn straw with 20% mulberry leaves; T60, 40% corn straw with 60% mulberry leaves; T80, 20% corn straw with 80% mulberry leaves. Values are means, and standard deviations are represented by vertical bars (n = 4). Bars with unlike letters indicate a significant difference (p<0.05). SOD, superoxide dismutase; GPX, glutathione peroxidase; CAT, catalase; MDA, malondialdehyde.

The inclusion of corn straw and mulberry leaves in the diet resulted in a notable enhancement of CAT activity in sheep’s liver (Figure 2C, p<0.05). Furthermore, the T60 and T80 groups exhibited significantly greater GPX activity compared to the Control group (Figure 2B, p<0.05). However, there were no significant differences in SOD activity or MDA content in the sheep liver (Figures 2A, 2D).

## DISCUSSION

Corn straw is well-known for its low CP content and poor digestibility, which can pose challenges in animal nutrition. In contrast, mulberry leaves are rich in CP, but also contain anti-nutritional factors. A recent *in vitro* study investigated rumen fluid fermentation by mixing corn straw and mulberry leaves in various ratios. Key parameters such as GP, VFAs, MCP, NH_3_-N, and IVOMD were measured. After comprehensive analysis, three groups with the most promising effects were identified based on the MFAEI and selected for animal feeding trial. The results demonstrated that the combination of corn straw and mulberry leaves significantly improved feed intake, muscle thickness and WHC in sheep. Additionally, it led to a decrease in BUN levels and enhanced antioxidant capabilities of the liver. Overall, this study underscores the potential benefits of combining corn straw with mulberry leaves for improving animal nutrition and overall health.

### Parameters of *in vitro* fermentation of corn straw and mulberry leaves

The GP level serves as an indicator of feed fermentability, a trait determined by both the degradation ability of rumen microorganisms and the characteristics of the substrate. It is generally observed that CP in the substrate is more readily fermented, whereas neutral detergent fiber (NDF) is less so. Research by de los Ángeles Bruni et al [21] highlighted a positive correlation between cumulative GP *in vitro* fermentation and CP content, while noting a negative correlation with the NDF content. In the current experiment, it was observed that the CP content of mulberry leaves exceeded that of corn straw, while the NDF content was lower. As the proportion of mulberry leaves in the substrate increased, the CP content rose, while the NDF content decreased, leading to a corresponding increase in cumulative GP. Similarly, findings from a study by Kang et al [22], which utilized varying proportions of pigeon pea and mulberry leaves as substrates for *in vitro* fermentation GP tests further supported these results by showing a rise in cumulative GP with an increase in the proportion of mulberry leaves.

The proportion and concentration of VFAs in rumen fluid are influenced by the composition of the diet. When dietary fiber content increases, the TVFA concentration in rumen fluid decreases, while the proportion of acetic acid increases [23]. Li et al [24] discovered a strong positive correlation between acetic acid concentration in rumen fluid and dietary NDF content. In our experiment, the proportion of mulberry leaves in the substrate increased, the NDF content decreased, and the concentrations of acetic acid and propionic acid initially increased and then decreased. Notably, the concentrations of acetic acid and propionic acid in the T60 and T80 groups surpassed those in the other groups. This can be attributed to the higher fermentability of mulberry leaves compared to corn straw, making it easier for microorganisms to decompose and utilize them. Consequently, mulberry leaves undergo rapid fermentation, resulting in the production of VFAs such as acetic acid and propionic acid, leading to higher concentrations of these acids as the proportion of mulberry leaves increases. Additionally, the synergistic effect of mulberry leaves and corn straw contributed to higher concentrations of acetic acid and propionic acid in the T60 and T80 groups compared to the T0 and T100 groups.

NH_3_-N in the rumen serves as the primary nitrogen source for the rumen microorganisms. An optimal NH_3_-N concentration can enhance the growth and reproduction of these microorganisms. However, excessive NH_3_-N concentration may lead to nitrogen wastage, while insufficient NH_3_-N concentration may hinder the activity of rumen microorganisms [25]. During the 6, 12, and 72 h time points, the NH_3_-N concentration in the T60, T80, and T100 groups was significantly higher compared to the other three groups. This elevated NH_3_-N concentration in these groups can be attributed to the higher protein content in the substrate, which is primarily degraded by rumen microorganisms, leading to increased NH_3_-N production. In conclusion, maintaining an appropriate NH_3_-N concentration is crucial for the optimal functioning of rumen microorganisms. The higher NH_3_-N concentrations observed in the T60, T80, and T100 groups highlight the importance of substrate protein content in promoting NH_3_-N production.

MCP is a crucial protein source for ruminants, meeting 40% to 80% of their protein requirements. The composition of the diet plays a significant role in MCP synthesis. Zhang et al [26] demonstrated that the levels of dietary carbohydrates and proteins, as well as their decomposition rates, influence the growth of rumen microorganisms, ultimately affecting the efficiency of MCP synthesis. In this experiment, increasing the proportion of mulberry leaves in the diet led to higher levels of high-quality protein in the substrate. This, in turn, increased MCP concentration.

García et al [27] discovered a strong positive correlation between IVDOM and CP, while the crude fiber content in feed is negatively correlated with digestibility. Similarly, Silva et al [28] found a positive relationship between IVDOM and cumulative GP. Our experiment showed that as the proportion of mulberry leaves increased, the CP content increased, NDF content decreased, and both cumulative GP and IVDOM also increased, consistent with previous research findings [27,28].

To assess the combined impact of multiple time points and multiple indices *in vitro* culture, the MFAEI can be utilized. This approach allows for a comprehensive quantification of all indices measured during *in vitro* fermentation, enabling the evaluation of synergistic effect between different diets. For calculation and analysis, cumulative GP, VFAs, NH_3_-N, MCP, and IVDOM were taken into account. The resultsof the analysis showed that the most considerable synergistic effect at 72 h was observed in group T60 (mulberry leaves to corn straw ratio of 60:40), while group T40 exhibited a negative synergistic effect. This negative effect may be attributed to the focus on MFAEI at 72 h, which could lead to an incidental imbalance in the results. However, this incidental negative effect does not significantly impact the overall test outcomes.

### Effects of the combination of corn straw and mulberry leaves on growth performances and meat quality of sheep

Although the feeding period was relatively short, an enhanced performance in sheep could still be observed with the combination of corn straw and mulberry leaves. The T60 group (40:60 ratio of corn straw to mulberry leaves) exhibited significantly higher feed intake compared to the Control group, leading to a slight increase in body weight gain. Furthermore, this combination improved abdominal fat deposition, with the T60 and T80 groups demonstrating greater rib and back muscle thickness compared to the Control group. In this study, the protein content of mulberry leaves was higher than that of corn straw. As the proportion of mulberry leaves increased in the diet, both dietary protein levels and MCP production also increased. Studies have shown that a protein diet occupies an important position in animal nutrition. Their nutritional value is not only reflected in suppling essential amino acids but also in their profound impact on animal growth, fattening and overall health [29,30]. First, proteins form the foundation of animal muscles and other important tissues, providing the amino acids necessary for animal growth, especially essential amino acids. Adequate protein intake during the growth and development of animals can effectively promotes muscle synthesis. Otherwise high-protein diets are typically associated with higher energy density, providing sufficient energy support for animals. The interaction between energy and protein is crucial for the fattening process of animals [31]. Studies have demonstrated that the oxidation of amino acids not only provides energy but also regulates insulin secretion and promotes fat synthesis [32].

Additionally, the combination of corn straw and mulberry leaf positively influenced the WHC of the LD muscle, resulting in reduced DL. The WHC of meat primarily depends on the structure of muscle fibers and the proportion of components within the muscle. Water in muscle cells primarily exist in the form of bound water, immobile water, and free water. The WHC of muscles predominantly refers to the retention of immobile water, which is influenced by the network structure of myofilament proteins and the amount of static electricity carried by these proteins [33]. Increasing the protein content in the diet can promote protein synthesis of in animal muscle cells, thereby enhanceing the WHC of muscles. Dietary supplementation of sepecific amino acids, such as valine and isoleucine, can improve the WHC of meat [34]. Moreover, certain nutrients in the diet, such as vitamin E, can exert antioxidant effects during the oxidation process of meat, slows down meat aging and maintaining its moisture and tenderness [35]. Mulberry leaves are rich in flavonoids, phenol acids and other beneficial components that posssess antioxidant properties [36]. These compounds can enhance the body’s antioxidant defenses, potentially aiding in improved muscle water retention.

Importantly, it was also showed that the BUN levels in sheep significantly decreased with the increase proportion of dietary mulberry leaves, indicating improved nitrogen utilization. BUN is an important index of the protein metabolism and its concentration is closely related to the nutritional status, nitrogen metabolism and diet composition of animals. Studies have shown that the higher-quality protein quality in the diet provides sufficient essential amino acids for animal growth, allowing ruminants to utilize these amino acids more effectively, thereby reducing ammonia production in the body and subsequently lowering urea synthesis and BUN levels [37,38]. Data analysis revealed that mulberry leaves not only contain a high content of CP but also exhibit a balanced amino acid profile, giving them a high biological value [39]. Additionally, diets rich in fermentable carbohydrates (such as cellulose and soluble sugars) and nitrogen-containing compounds (CP) can improve protein synthesis by rumen microorganisms, thereby reducing the ruminant’s need for self-protein degradation and decreasing urea production [40]. These findings indicate that the combination of corn straw and mulberry leaves can provide an optimal carbon and nitrogen source for rumen microbial protein synthesis.

### Antioxidative effect of corn straw combined with mulberry leaves

The enhanced antioxidant capacity of the liver plays a crucial role in livestock management, as oxidative stress can lead to a range of health issues such as compromised immune function and decreased productivity [41]. The notable increase in DPPH and ABTS scavenging capabilities indicated that the combined diet of corn straw and mulberry leaves effectively provided enhanced protection to the liver tissues of the sheep. Interestingly, this improvement in free radical scavenging ability and antioxidant enzyme activity appeared to be positively associated with a higher proportion of mulberry leaves in the diet. Studies suggest that mulberry leaves are rich in various antioxidant compounds [36], which likely contribute to the observed enhancements in oxidative stress markers in the liver. Ouyang et al [42] demonstrated that mulberry leaves could upregulate the gene expression of antioxidant-related enzymes in the liver of Hu sheep, thereby improving the antioxidant capacity of animals. However, our results showed no significant impact of the corn straw and mulberry leaf combination on the levels of ROS and MDA in the liver. This lack of effect may indicate that the test subjects were relatively healthy and that the dietary combination did not induce oxidative stress under the experimental conditions. This is encouraging, as it suggests that the sheep were able to maintain homeostasis and effectively manage oxidative species in a healthy state, supporting the overall health benefits of this diet. Furthermore, our findings imply that incorporating 40% corn straw and mulberry leaves into the diet represents a reasonable proportion for maximizing health benefits without adverse effects. This combination not only provides nutritional value but also enhances the health profile of the sheep through improved antioxidant defenses.

## CONCLUSION

Our findings demonstrated that the mixture of corn straw and mulberry leaves had a positive synergistic effect and the maximum associative effect indexes were found in the 60:40 combination. Under this synergistic effect, corn straw and mulberry leaves improved feed intake, muscle thickness, muscle water retention and liver antioxidant capacity in sheep. The implications of these findings are significant, as they provide a sustainable solution for optimizing sheep diets. Additionally, this approach offers a practical method to utilize agricultural waste and reduce breeding costs.

## Figures and Tables

**Table 1:** Nutritional levels of various combinations of corn straw and mulberry leaves on an air-dry basis (%)

Items	Groups^1)^					

	T0	T20	T40	T60	T80	T100
DM	89.07	89.22	89.38	89.53	89.69	89.84
CP	5.52	8.49	11.46	14.43	17.40	20.37
EE	6.30	6.67	7.05	7.42	7.81	8.18
Ash	4.34	5.00	5.64	6.29	6.93	7.59
NDF	73.92	66.02	58.11	50.22	42.31	34.41
ADF	38.84	34.33	29.84	25.33	20.84	16.33

1) T0, 100% corn straw; T20, 80% corn straw with 20% mulberry leaves; T40, 60% corn straw with 40% mulberry leaves; T60, 40% corn straw with 60% mulberry leaves; T80, 20% corn straw with 80% mulberry leaves; T100, 100% mulberry leaves.

DM, dry matter; CP, crude protein; EE, ether extract; NDF, neutral detergent fiber; ADF, acid detergent lignin.

**Table 2:** Ingredients and chemical composition of experimental diets

Items	Groups^1)^			

	Control	T20	T60	T80
Ingredients (%)				
Corn straw	40	32	16	8
Mulberry leaves	0	8	24	32
Cracked corn	40.5	38.5	40.5	43.8
Wheat flour	0	4.5	6.9	10.8
Cardamom	18	15.5	11.1	3.9
Salt	0.5	0.5	0.5	0.5
Premix	1	1	1	1
Chemical composition				
ME (MJ/kg)	10.11	10.10	10.19	10.19
CP (%)	13.36	13.99	14.95	13.82
NDF (%)	35.48	33.43	27.44	24.93
ADF (%)	18.68	17.11	13.34	11.39
EE (%)	4.17	4.38	4.80	5.09

1) Control, 100% corn straw; T20, 80% corn straw with 20% mulberry leaves; T60, 40% corn straw with 60% mulberry leaves; T80, 20% corn straw with 80% mulberry leaves.

ME, metabolizable energy; CP, crude protein; NDF, neutral detergent fiber; ADF, acid detergent fiber; EE, ether extract.

**Table 3:** Effects of varying proportions of corn straw and mulberry leaves on the gas production parameters of 72 h *in vitro* culture solution^1)^

Items	Groups^2)^						SEM	p-value

	T0	T20	T40	T60	T80	T100		
Theoretical maximum GP (mL/g DM)	58.41^d^	56.95^d^	60.51^d^	69.94^c^	74.51^b^	82.58^a^	2.03	0.005
Potential GP (mL/g DM)	65.37^c^	62.81^c^	64.13^c^	71.64^b^	73.69^b^	82.20^a^	1.37	0.007
Constant of GP rate (mL/h)	0.047^e^	0.056^d^	0.082^c^	0.106^a^	0.095^b^	0.103^ab^	0.05	0.003
Cumulative GP (mL/g DM)	63.75^d^	64.19^d^	65.69^cd^	70.74^bc^	72.28^b^	84.06^a^	1.49	0.006

1) Each value represents the mean±SEM of 7 replicates (n = 7).

2) T0, 100% corn straw; T20, 80% corn straw with 20% mulberry leaves; T40, 60% corn straw with 40% mulberry leaves; T60, 40% corn straw with 60% mulberry leaves; T80, 20% corn straw with 80% mulberry leaves; T100, 100% mulberry leaves.

a–e Different letters in the same line indicate a significant difference between treatments (p<0.05).

SEM, standard error of the mean; GP, gas production; DM, dry matter.

**Table 4:** Effects of varying proportions of corn straw and mulberry leaves on the VFAs concentration of 72 h *in vitro* culture solution^1)^

Items	Time/h	Groups^2)^						SEM	p-value

		T0	T20	T40	T60	T80	T100		
TVFA (mmol/L)	0	27.77	27.77	27.77	27.77	27.77	27.77	0.98	1.000
	3	39.32^b^	45.54^a^	45.85^a^	44.31^ab^	46.04^a^	39.86^b^	1.01	0.008
	6	40.04^c^	45.37^ab^	47.57^a^	39.92^c^	37.89^c^	41.25^bc^	1.21	0.007
	12	45.82^c^	44.43^cd^	38.11^d^	58.11^b^	71.17^a^	60.53^b^	2.35	0.004
	24	54.21^d^	47.02^e^	58.45^d^	89.35^a^	79.64^b^	70.93^c^	3.38	0.001
	48	59.25^d^	49.05^e^	61.85^d^	89.17^b^	95.34^a^	85.56^c^	3.50	0.003
	72	72.34^b^	80.95^b^	77.11^b^	96.51^a^	100.10^a^	100.73^a^	3.38	0.007
Acetate (mmol/L)	0	20.88	20.88	20.88	20.88	20.88	20.88	0.41	1.000
	3	24.94^b^	26.81^ab^	27.42^ab^	28.62^ab^	29.07^a^	27.17^ab^	0.53	0.024
	6	27.11^bc^	30.32^ab^	31.67^a^	26.52^bc^	25.37^c^	28.17^abc^	0.77	0.008
	12	30.34^c^	29.50^c^	26.79^c^	43.99^b^	53.40^a^	42.31^b^	1.93	0.007
	24	31.31^e^	30.52^e^	39.60^d^	65.21^a^	55.52^b^	50.96^c^	2.62	0.003
	48	38.63^c^	35.18^d^	34.20^d^	64.60^a^	65.20^a^	58.95^b^	2.62	0.005
	72	43.18^c^	51.65^b^	42.72^c^	71.44^a^	74.91^a^	70.76^a^	3.23	0.005
Propionate (mmol/L)	0	5.31	5.31	5.31	5.31	5.31	5.31	0.40	1.000
	3	11.91^c^	13.81^b^	15.75^a^	11.77^c^	12.69^bc^	10.13^d^	0.47	0.004
	6	10.48^c^	12.22^ab^	12.92^a^	11.59^abc^	10.26^c^	11.19^bc^	0.39	0.008
	12	12.50^b^	11.96^b^	9.65^c^	12.84^b^	15.13^a^	14.68^a^	0.44	0.007
	24	13.55^c^	13.25^c^	14.19^c^	18.66^ab^	20.95^a^	15.40^bc^	0.86	0.008
	48	15.62^d^	9.53^e^	23.49^b^	18.76^c^	26.04^a^	22.55^b^	1.18	0.003
	72	24.49^b^	24.98^b^	29.02^a^	19.51^c^	18.96^c^	24.26^b^	0.90	0.007
Butyrate (mmol/L)	0	1.59	1.59	1.59	1.59	1.59	1.59	0.19	1.000
	3	2.47^b^	4.93^a^	2.69^b^	3.93^a^	4.28^a^	2.56^b^	0.24	0.009
	6	2.45^b^	2.83^ab^	2.97^a^	1.82^d^	2.25^bc^	1.88^cd^	0.12	0.002
	12	2.98^b^	2.97^b^	2.67^bc^	2.28^c^	2.63^bc^	3.54^a^	0.11	0.008
	24	5.35^a^	3.23^b^	4.68^a^	5.48^a^	3.17^b^	4.58^a^	0.22	0.007
	48	5.00^b^	4.34^bc^	4.15^c^	5.80^a^	4.10^c^	4.06^c^	0.17	0.008
	72	4.68^bc^	4.32^c^	5.37^ab^	5.56^ab^	6.23^a^	5.71^a^	0.16	0.024
Acetate/propionate	0	4.00	4.00	4.00	4.00	4.00	4.00	0.22	1.000
	3	2.10^bc^	1.95^cd^	1.77^d^	2.45^ab^	2.30^b^	2.70^a^	0.08	0.013
	6	2.60	2.47	2.30	2.28	2.48	2.52	0.16	0.089
	12	2.44^c^	2.48^c^	2.80^b^	3.43^a^	3.54^a^	2.91^b^	0.08	0.007
	24	2.37^b^	2.38^b^	2.80^b^	3.54^a^	2.75^b^	3.32^a^	0.11	0.009
	48	2.48^b^	3.71^a^	1.46^c^	3.46^a^	2.53^b^	2.66^b^	0.14	0.007
	72	1.77^cd^	2.10^c^	1.47^d^	3.69^a^	3.97^a^	2.98^b^	0.18	0.015

1) Each value represents the mean±SEM of 3 replicates (n = 3).

2) T0, 100% corn straw; T20, 80% corn straw with 20% mulberry leaves; T40, 60% corn straw with 40% mulberry leaves; T60, 40% corn straw with 60% mulberry leaves; T80, 20% corn straw with 80% mulberry leaves; T100, 100% mulberry leaves.

a–e Different letters in the same line indicate a significant difference between treatments (p<0.05).

VFAs, volatile fatty acids; SEM, standard error of the mean; TVFA, total volatile fatty acids.

**Table 5:** Effects of varying proportions of corn straw and mulberry leaves on the pH, and NH3-N, MCP, IVOMD concentration of 72 h *in vitro* culture solution^1)^

Items\	Time/h	Groups^2)^						SEM	p-value

		T0	T20	T40	T60	T80	T100		
pH	0	7.15	7.15	7.15	7.15	7.15	7.15	0.02	1.000
	3	7.08	7.07	7.06	7.07	7.06	7.05	0.03	0.933
	6	7.06	7.06	7.07	7.04	7.02	7.01	0.03	0.064
	12	7.05	7.05	7.03	6.99	7.04	7.02	0.06	0.252
	24	7.03	7.03	7.01	7.00	6.98	6.96	0.07	0.074
	48	7.00	6.99	6.96	6.87	6.81	6.82	0.09	0.129
	72	6.88	6.85	6.85	6.76	6.74	6.75	0.12	0.139
NH_3_-N (mg/dL)	0	6.40	6.40	6.40	6.40	6.40	6.40	0.12	1.000
	3	6.57^b^	7.14^ab^	7.38^a^	7.73^a^	7.70^a^	7.40^a^	0.27	0.009
	6	8.31^b^	8.98^b^	8.35^b^	14.47^a^	13.61^a^	14.27^a^	0.73	0.008
	12	9.13^b^	9.82^b^	9.43^b^	13.68^a^	13.51^a^	14.67^a^	0.52	0.008
	24	13.82^e^	15.00^d^	14.54^de^	20.11^a^	18.72^b^	15.87^c^	0.35	0.003
	48	18.20^c^	19.28^c^	18.92^c^	28.65^b^	32.68^a^	30.18^ab^	1.64	0.008
	72	18.68^c^	22.59^b^	23.30^b^	40.94^a^	41.57^a^	41.87^a^	1.02	0.007
MCP (mg/mL)	0	2.74	2.74	2.74	2.74	2.74	2.74	0.01	1.000
	3	2.43^e^	2.81^c^	2.56^d^	3.41^b^	3.56^a^	3.37^b^	0.09	0.002
	6	2.27^bc^	2.36^b^	2.28^bc^	2.25^c^	2.37^b^	2.88^a^	0.05	0.009
	12	2.08^c^	2.17^b^	1.94^d^	3.19^a^	3.28^a^	3.19^a^	0.14	0.008
	24	2.02^d^	1.88^e^	1.92^e^	3.23^b^	3.08^c^	3.37^a^	0.14	0.002
	48	1.63^b^	1.61^b^	1.66^b^	2.61^a^	2.74^a^	2.70^a^	0.11	0.007
	72	1.07^d^	1.41^c^	1.37^c^	2.17^a^	1.92^b^	2.15^a^	0.08	0.001
IVOMD (%)	3	0.12^e^	0.16^d^	0.19^c^	0.21^b^	0.25^a^	0.27^a^	0.01	0.003
	6	0.15^e^	0.20^d^	0.23^c^	0.23^c^	0.27^b^	0.32^a^	0.01	0.003
	12	0.22^c^	0.29^b^	0.41^a^	0.28^b^	0.42^a^	0.41^a^	0.02	0.007
	24	0.38^f^	0.48^e^	0.58^d^	0.64^c^	0.71^b^	0.79^a^	0.03	0.001
	48	0.49^e^	0.58^d^	0.66^c^	0.69^c^	0.83^b^	0.94^a^	0.04	0.003
	72	0.54^f^	0.67^e^	0.72^d^	0.78^c^	0.87^b^	0.96^a^	0.03	0.001

1) Each value represents the mean±SEM of 3 replicates (n = 3).

2) T0, 100% corn straw; T20, 80% corn straw with 20% mulberry leaves; T40, 60% corn straw with 40% mulberry leaves; T60, 40% corn straw with 60% mulberry leaves; T80, 20% corn straw with 80% mulberry leaves; T100, 100% mulberry leaves.

a–f Different letters in the same line indicate a significant difference between treatments (p<0.05).

NH_3_-N, ammonia nitrogen; MCP, microbial crude protein; IVOMD, *in vitro* organic matter digestibility; SEM, standard error of the mean.

**Table 6:** Associative effect indexes of different ratios of corn straw and mulberry leaves at 72 h in vitro culture solution^1)^

Groups^2)^	SFAEI					MFAEI

	Cumulative GP	TVFA	MCP	NH3-N	IVOMD	
T20	−5.34	3.75	9.64	−3.12	7.37	12.30
T40	−8.60	−7.86	−8.78	−16.65	1.69	−40.20
T60	−6.84	7.98	26.31	25.60	−1.51	51.54
T80	−9.64	5.31	−0.72	11.65	−0.68	5.92

1) Each data was calculated from the mean value of fermentation parameters at 72 h.

2) T20, 80% corn straw with 20% mulberry leaves; T40, 60% corn straw with 40% mulberry leaves; T60, 40% corn straw with 60% mulberry leaves; T80, 20% corn straw with 80% mulberry leaves.

SFAEI, single-factor associative effect index; GP, gas production; TVFA, total volatile fatty acids; MCP, microbial crude protein; NH_3_-N, ammonia nitrogen; IVOMD, *in vitro* organic matter digestibility; MFAEI, multiple-factors associative effect index.

**Table 7:** Effects of varying proportions of corn straw and mulberry leaves on growth performance and slaughter of sheep^1)^

Items	Groups^2)^				SEM	p-value

	Control	T20	T60	T80		
Growth performance						
Initial LW (kg)	24.92	25.30	24.91	25.46	0.363	0.760
Final LW (kg)	38.92	39.17	39.22	40.02	0.344	0.295
ADG (kg)	13.99	13.87	14.30	14.56	0.381	0.508
ADFI (g)	1,174^b^	1,220^ab^	1,260^a^	1,222^ab^	17.82	0.040
F/G	4.72	4.94	4.90	4.77	0.146	0.932
Carcass trait						
Alive weight (kg)	38.32	38.28	38.25	38.98	0.291	0.153
Carcass weight (kg)	17.02	17.15	17.58	16.90	0.282	0.737
Eye muscle area (cm^2)^	23.23	23.86	23.32	23.95	0.549	0.815
Subcutaneous fat (g)	932.0	836.5	1025.0	933.0	43.12	0.493
Abdominal fat (g)	178.8	254.5	232.2	320.0	30.00	0.090
Pericardial fat (g)	40.49	35.13	46.37	30.04	3.829	0.699
Perirenal fat (g)	60.69	89.92	88.96	82.54	6.812	0.297
Tail fat (g)	648.5	711.8	557.2	580.0	79.20	0.544
Pot fat (g)	27.14	18.44	17.22	29.88	3.281	0.553
Rib thickness	0.440^b^	0.507^ab^	0.535^a^	0.556^a^	0.024	0.036
Back thickness	0.364^bc^	0.318^c^	0.426^a^	0.452^a^	0.030	0.004

1) Growth performance data value represents the mean±SEM of 8 replicates (n = 8), and carcass trait data value represents the mean±SEM of 4 replicates (n = 4).

2) Control, 100% corn straw; T20, 80% corn straw with 20% mulberry leaves; T60, 40% corn straw with 60% mulberry leaves; T80, 20% corn straw with 80% mulberry leaves.

a–c Different letters in the same line indicate a significant difference between treatments (p<0.05).

LW, live weight; ADG, average daily gain; ADFI, average daily feed intake; F/G, ADFI/ADG.

**Table 8:** Effects of varying proportions of corn straw and mulberry leaves on meat quality of sheep^1)^

Items		Groups^2)^				SEM	p-value

		Control	T20	T60	T80		
Meat color							
L*							
	LD	37.50	34.95	35.68	34.14	0.179	0.667
	AL	40.18	38.61	41.22	40.60	0.265	0.874
	HL	36.97	36.03	35.88	36.08	0.190	0.959
a*							
	LD	15.19	14.76	15.54	16.23	0.031	0.908
	AL	15.12	16.88	17.43	15.12	0.073	0.523
	HL	15.22	17.65	18.51	18.47	0.073	0.227
b*							
	LD	3.755	4.862	5.260	5.172	0.053	0.441
	AL	6.505	5.662	6.722	5.540	0.025	0.877
	HL	5.145	5.708	6.085	6.462	0.081	0.310
WHC (%)							
	LD	12.35^c^	22.60^a^	17.35^b^	16.72^b^	0.021	<0.001
	AL	12.10^c^	23.43^a^	17.00^b^	13.55^c^	0.025	0.004
	HL	13.65^c^	21.55^a^	18.15^b^	14.27^c^	0.018	<0.001
DL (%)							
	LD	20.10^a^	12.75^b^	9.53^b^	12.53^b^	0.022	<0.001
	AL	12.45	8.75	6.58	10.13	0.012	0.259
	HL	6.58	8.28	6.38	7.70	0.005	0.456
CL (%)							
	LD	29.18^a^	21.55^b^	32.10^a^	29.63^a^	0.023	<0.001
	AL	29.18	30.37	33.88	32.53	0.013	0.573
	HL	36.45^a^	31.22^b^	32.78^ab^	29.95^b^	0.014	0.069
WBSF							
	LD	36.00	38.52	42.41	49.49	0.505	0.568
	AL	46.39	44.58	46.24	51.26	0.632	0.923
	HL	50.98	43.99	40.73	39.33	0.200	0.860

1) Each value represents the mean±SEM of 4 replicates (n = 4).

2) Control, 100% corn straw; T20, 80% corn straw with 20% mulberry leaves; T60, 40% corn straw with 60% mulberry leaves; T80, 20% corn straw with 80% mulberry leaves.

a–c Different letters in the same line indicate a significant difference between treatments (p<0.05).

SEM, standard error of the mean; LD, *longissimus dorsi*; AL, anterior leg; HL, hind leg; WHC, water-holding capacity; DL, drip loss; CL, cooking loss; WBSF, Warner-Bratzler shear force.

**Table 9:** Effects of varying proportions of corn straw and mulberry leaves on hematology analysis of sheep^1)^

Items	Groups^2)^				SEM	p-value

	Control	T20	T60	T80		
ALT (U/L)	18.17^a^	18.67^a^	15.33^b^	13.17^c^	0.286	0.012
AST (U/L)	105.17^a^	100.67^a^	82.83^b^	84.67^b^	1.621	0.010
AST/ALT	6.043	5.558	6.113	6.566	0.381	0.498
TP (g/L)	56.50	56.37	57.75	55.38	0.485	0.776
Alb (g/L)	23.48	24.10	23.77	23.22	0.254	0.589
Glob (g/L)	33.02	32.27	33.98	32.17	0.467	0.996
A/G	0.71	0.75	0.70	0.72	0.014	0.771
CR (μmol/L)	47.83	47.33	48.33	52.17	1.109	0.173
BUN (mmol/L)	8.88^a^	6.37^b^	6.87^b^	5.87^c^	0.268	0.012
UA (μmol/L)	13.33	14.17	10.17	8.83	0.661	0.095
GLU (mmol/L)	3.46	3.53	3.52	3.49	0.058	0.896
CHO (mmol/L)	1.60	1.46	1.39	1.41	0.047	0.099
TG (mmol/L)	0.32	0.36	0.46	0.41	0.030	0.124
HDL-C (mmol/L)	0.76	0.68	0.68	0.70	0.022	0.387
LDL-C (mmol/L)	0.54	0.46	0.44	0.44	0.024	0.064

1) Each value represents the mean±SEM of 4 replicates (n = 4).

2) Control, 100% corn straw; T20, 80% corn straw with 20% mulberry leaves; T60, 40% corn straw with 60% mulberry leaves; T80, 20% corn straw with 80% mulberry leaves.

a–c Different letters in the same line indicate a significant difference between treatments (p<0.05).

SEM, standard error of the mean; ALT, alanine aminotransferase; AST, aspartate aminotransferase; TP, total protein; Alb, albumin; Glob, globulin; CR, creatinine; BUN, blood urea nitrogen; UA, uric acid; GLU, glucose; CHO, total cholesterol; TG, triglyceride; HDL-C, high-density lipoprotein cholesterol; LDL-C, low density lipoprotein cholesterol.
